# Chromatin accessibility and gene expression in the parasite Trichomonas vaginalis

**DOI:** 10.21203/rs.3.rs-5455511/v1

**Published:** 2024-12-16

**Authors:** Agustina Prat, Daniela Muñoz, Ayelen Lizarraga, Julieta Seifert-Gorzycki, Estefania Sanchez-Vazquez, Patrica Johnson, Pablo Hernan Strobl Mazzulla, Natalia de Miguel

**Affiliations:** Instituto Tecnológico Chascomús (INTECH), CONICET-UNSAM; Instituto Tecnológico Chascomús (INTECH), CONICET-UNSAM; Instituto Tecnológico Chascomús (INTECH), CONICET-UNSAM; Instituto Tecnológico Chascomús (INTECH), CONICET-UNSAM; Instituto Tecnológico Chascomús (INTECH), CONICET-UNSAM; University of California, Los Angeles; Instituto Tecnológico Chascomús (INTECH), CONICET-UNSAM; Instituto Tecnológico Chascomús (INTECH), CONICET-UNSAM

**Keywords:** Parasite, Trichomonas, gene expression, epigenetic, chromatin, pathogenesis

## Abstract

*Trichomonas vaginalis*, the most common non-viral sexually transmitted parasite, causes more than 270 million infections annually. The infection’s outcome varies greatly depending on different factors that include variation in human immune responses, the vaginal microbiome, and the inherent virulence of the strain. Although the pathogenicity of the different strains depends, at least partially, on differential gene expression of virulence genes; the regulatory mechanisms governing this transcriptional control remain incompletely understood. While many studies have reported a positive correlation between gene expression and chromatin accessibility in other cells, this relationship has not been analyzed in *T. vaginalis*. To address these questions, we selected two contrasting *T. vaginalis* strains based on their interactions with host cells: B7268 strain, a highly adherent one and resistant to metronidazole, and NYH209 strain, a poorly adherent one and sensitive to metronidazole. Next, we combined the assay for transposase-accessible chromatin using sequencing (ATAC-seq) with RNA sequencing (RNA-seq), to delve into the relationship between chromatin accessibility and gene expression in these distinct *T. vaginalis* strains. Our findings demonstrate a correlation between chromatin accessibility and gene expression across both strains. Moreover, we found that chromatin accessibility plays a pivotal role in modulating mRNA expression levels of several established genes linked to parasite pathogenesis and drug resistance. We also identified several open chromatin peaks residing at intergenic regions, revealing possible distal regulatory elements that may control gene expression. These results highlight the importance of chromatin accessibility in modulating gene expression in the parasite *T. vaginalis*, with possible consequences in pathogenesis and/or drug treatment.

## Background

*Trichomonas vaginalis* is a common sexually transmitted parasite that colonizes the human urogenital tract causing infections that range from asymptomatic to highly inflammatory. Worldwide prevalence of trichomoniasis (over 276 million cases annually) surpasses that of gonorrhea, syphilis, and chlamydia [1]. Chronic infections can lead to severe complications such as infertility, premature birth during pregnancy, increases the risk of acquiring HIV, cervical cancer and aggressive prostate cancer [2]. The infection’s outcome exhibits significant variability due to variation in human immune responses, indigenous vaginal microbes, and the inherent virulence of the strain [3]. Specifically, variability in disease manifestation may be attributed to numerous parasite strains circulating in the population [4], each displaying varying degrees of adherence and cytolysis towards host cells [5]. Similarly, a great variation in the level of drug resistance has been described for the different isolated *T. vaginalis* strains around the world [6]. To treat infection, metronidazole (MTZ) and tinidazole are the only two effective drugs approved by the United States Food and Drug Administration [7]. Clinical failure of MTZ treatment ranges from ∼4% in the US [8] to 17% in Papua New Guinea [9]. The development of drug resistance in trichomonads has been linked to the altered expression of key enzymes involved in energy production and antioxidant defense mechanisms, which in turn affects the activation of 5-nitroimidazole drugs [7]. Specifically, resistant strains have been reported to exhibit reduced expression of enzymes such as pyruvate oxidoreductase, ferredoxin, nitroreductase, hydrogenase, thioredoxin reductase, and flavin reductase [7]. Differential gene expression is also a key factor in the pathogenicity of various *T. vaginalis* strains, as demonstrated by differences in the abundance of surface proteins across strains with distinct pathogenic phenotypes [10]. Although the control of gene expression seems to be important for parasite pathogenesis and drug treatment resistance [6, 10], it is still largely unknown how these changes in transcriptional profiles are controlled as very few transcriptional regulatory elements have been described until now [11]. Our recent works highlighted the importance of epigenetics and chromatin in the regulation of transcription [12]. Specifically, we demonstrated that histone acetylation (H3Kac) is a permissive histone modification that functions to mediate chromatin accessibility, gene transcription and pathogenesis of the parasite *T. vaginalis* [13, 14]. Additionally, a new role for 6mA in modulating 3D chromatin structure and gene expression in this parasite has been demonstrated [15]. Epigenetic signatures can be revealed from genome-wide mapping of chromatin accessibility. Differences in chromatin accessibility at regulatory regions are anticipated to mirror the transcriptional activity within a particular cell or under specific conditions by influencing transcription factor binding. The development of the assay for transposase-accessible chromatin with high-throughput sequencing (ATAC-seq), which employs the transposase Tn5 to fragment chromatin and integrate adapters for next generation sequencing into open chromatin regions, provides information on open chromatin, nucleosome positioning, thereby enabling conclusions on functional consequences [16]. Several studies that have integrated ATAC-seq and RNA-seq data reported a positive correlation between the chromatin accessibility signal at a gene’s promoter and its expression [17, 18]. Despite this correlation, many studies found that changes in the chromatin accessibility are not always associated with expected changes in transcription [17, 19]. To better understand the role of chromatin accessibility in modulation of gene expression, we generated ATAC-seq data to map chromatin accessibility and RNA-seq analysis to profile gene expression in two *T. vaginalis* strains with different phenotypes: a highly adherent, cytolytic and resistant to metronidazole strain (B7268) as well as a poorly adherent, non-cytolytic and sensitive to metronidazole *T. vaginalis* strain (NYH209). An integrated analysis combining RNA-seq and ATAC-seq datasets, illustrated the impact of chromatin accessibility on gene expression modulation as a positive correlation between chromatin accessibility at the promoter as well as gene coding regions with the corresponding levels of gene expression was observed in both strains. Implying a potential role of chromatin accessibility in shaping the strain’s phenotype, we identified a distinct set of genes exhibiting differential chromatin accessibility that aligned with differential mRNA expression levels in each strain. Pathway analysis of these differentially expressed genes revealed functions linked to the pathogenesis in the B7268 strain. These results demonstrate for the first time an intimate involvement of the chromatin accessibility in regulation of gene expression in the parasite *T. vaginalis*.

## Methods

### Parasite culture.

*Trichomonas vaginalis* strains B7268 [20] and NYH209 (ATCC 50146) were cultured in tubes under microaerophilic conditions at 37°C in TYM medium (tryptose, yeast extract, and maltose) [21] supplemented with 10% horse fetal serum, 10 U/ml penicillin, and 10 µg/ml streptomycin (Invitrogen).

### RNA-sequencing.

Total RNA was extracted from ~ 3.5 × 10^6^ *T. vaginalis* (NYH209 and B7268 strains) according to the protocol outlined in the illustra^™^ RNAspin Mini RNA Isolation Kit (GE Healthcare, UK) following the manufacturer’s instructions. RNA quality and quantity were assessed using an Agilent Bioanalyzer with RNA integrity numbers (RINs) of > 7 for all samples. The mRNA libraries were paired-end (100 bp) sequenced with Illumina using TruSeq Stranded mRNA (Macrogen, Inc).

### Bioinformatics Analysis of RNA-seq Data.

After sequencing, ~ 20 million reads were generated per RNA-seq library. The software FastQC [22] was used for quality control of the sequencing. The adapter sequences content was identified and trimmed using Trimmomatic [23]. Then, HISAT2 [24] was used to align the RNA-seq data sets to the G3 2022 reference genome sequence [25], and the results showed an overall alignment rate of > 90% for all libraries. In order to quantify the counts, featureCounts [26] was used with default paired-end parameters. Principal component analysis was carried out to evaluate the variation through biological replicates. The expression level of each transcript was quantified as FPKM (fragments per kilobase of exon per million fragments mapped) using Stringtie [27]. Differential gene expression was explored using DEseq2 package, P-values were adjusted using p-value adjust (padj) (26). Only changes where | log2-fold change | ≥ 1 and padj < 0.05 were considered significant.

### ATAC-seq.

ATAC-seq assays (NYH209 and B7268 strains) were performed by Quick Biology, Inc. Briefly, nuclei isolation was performed from 2 million cells from each strain, then, samples were exposed to the Tn5 transposase (Illumina) for 30 minutes at 37°C. Tn5 transposase acts as a homodimer to simultaneously fragment the chromatin and insert the necessary adapters for downstream amplification and sequencing [28]. Immediately after incubation with Tn5 the samples were purified using a commercial purification kit. This was followed by a 12-cycle PCR to amplify the DNA fragments and a second round of purification. Finally, the libraries were quantified and sequenced in paired-end read mode using the Illumina HiSeq X platform.

### Bioinformatics Analysis of ATAC-seq Data.

After sequencing, ~ 20 million reads were generated per ATAC-seq library. For quality control of pairwise sequencing data, FastQC software [22] was used. The adaptor sequence content was identified and trimmed using Trimmomatic [23]. High-quality adapter-free reads were aligned to the G3 *T. vaginalis* reference genome [25] with Bowtie2 using -X 2000, -3 10 and all other parameters predetermined by the program [29]. The resulting BAM files were filtered for quality (> Q10) and used as input files in the MACS2 program, which was run with the parameters -f BED, -g 1.8e8, --shift – 100 and --extsize 200 [30]. BAM, BED and index files were then imported into RStudio (version 4.3.1). Differentially Accessible Regions (DARs) were determined using DiffBind with the parameter “minOverlap = 2” and DeSeq2 (within DiffBind) considering log2-fold change | ≥ 1 and FDR < 0.05 as significant [31]. The peaks were annotated using the *T. vaginalis* reference genome using HOMER (annotatePeaks.pl). The accessibility level of each region was quantified as CPM (counts per million) using ¨score = DBA_SCORE_TMM_READS_EFECTIVE_CPM¨ [31]. Profiles of ATAC-seq peaks, including density heatmaps and average profiles across the defined intervals in both strains were generated using the deepTools plotHeatmap function in Galaxy Bioinformatics [32].

### Analysis of Motif enrichment.

To identify enriched motifs in accessible intergenic regions, we utilized the HOMER software suite [33]. Initially, we defined the intergenic regions using the genomic coordinates obtained from ATAC-seq data. These coordinates were processed with BEDtools [34] to filters regions with at least 80% overlap with the intergenic intervals, using the parameters -f 0.8, -F 0.8 and -e for exact matches. All motif analyses were performed using HOMER’s FindMotifGenome.pl [33] tool with the parameters of ‘-size 500’.

### Combining RNA and ATAC sequencing.

Each gene was classified according to its level of expression and accessibility. Considering the ATAC-seq data, genes were classified as low accessible (LA) if the log2(CPM + 1) below the 40th percentile and as highly accessible (HA) if the log2(CPM + 1) was greater than the 60th percentile of the data. Similarly, genes were classified as low expression (LE) if the log2(FPKM + 1) was below the 40th percentile and as highly expressed (HE) if their log2(FPKM + 1) was higher than the 60th percentile of the data. Then, four categories were defined: HA–HE (high accessibility and expression), HA–LE (high accessibility and low expression), LA–LE (low accessibility and expression) or LA–HE (low accessibility and high expression).

### Chromatin accessibility and gene expression differential data.

To classify each gene based on its differential expression (DEG) and chromatin accessibility (DAR), RNA-seq and ATAC-seq datasets were integrated. For this purpose, genes were classified as differentially accessible (DAR) if they exhibited a log-fold change (log2FC) in chromatin accessibility that met the threshold for significance (| log2-fold change | ≥ 1 and FDR < 0.05). Similarly, genes were classified as differentially expressed (DEG) if their expression levels showed significant changes (| log2-fold change | ≥ 1 and padj < 0.05). Then, to visualize the relationship between DAR and DEG a scatter plot was created, where each gene was represented by plotting its log2 fold change (log2FC) in chromatin accessibility on the Y-axis and log2FC in gene expression on the X-axis.

### Gene ontology analysis.

The gene ontology enrichment analysis was performed with the Results Analysis option of TrichDB (http://TrichDB.org) using the default parameters [35].

## Results

### Differences in gene expression between phenotypically different T. vaginalis strains

**Figure 1:**
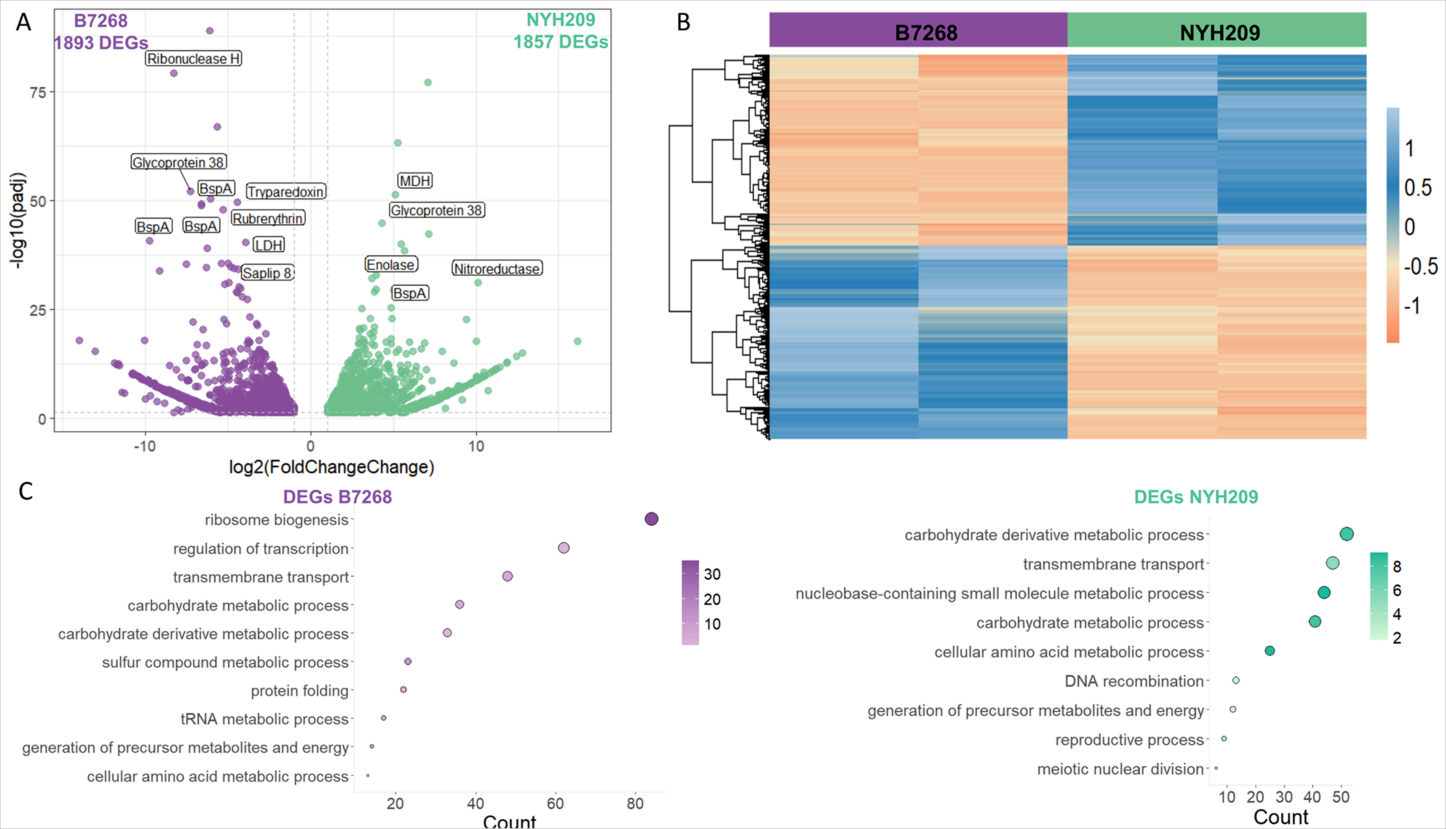
RNA-seq analysis. (A) Volcano plot showing differentially expressed genes in two *T. vaginalis*strains: NYH209 and B7268. Each dot represents a gene, with purple and green dots indicating statistically significant differentially expressed genes (DEGs) in NYH209 and B7268 strain, respectively (|log2-fold change| ≥ 1 and padj < 0.05). Genes highlighted in the plot include TVAGG3_0191610 (BspA), TVAGG3_0664570 (Ribonuclease H), TVAGG3_0408780 (Glycoprotein 38), TVAGG3_0387950 (BspA), TVAGG3_0387970 (BspA), TVAGG3_0349970 (Rubrerythrin), TVAGG3_0061400 (Saplip 8), TVAGG3_0961250 (Tryparedoxin), TVAGG3_0776680 (LDH), TVAGG3_0309560 (Enolase), TVAGG3_0430560 (Glycoprotein 38), TVAGG3_0300500 (BspA), TVAGG3_0341660 (MDH), TVAGG3_0959810 (Nitroreductase). (B) Heatmap of DEGs ordered by decreased log2-fold change per group identified from DESeq2. Colors indicate the normalized count z-score in each B7268 DEG across NYH209. (C) Differentially expressed genes (DEGs) in *T. vaginalis* strain NYH209 and B7268, categorized by biological processes. Color represents enrichment significance, and size of the bubble represents the number of DEG enriched in the pathway.

To evaluate if differences in gene expression could play a role in shaping phenotypic distinctions among *T. vaginalis* strains [10], we performed RNA-seq on two parasite strains displaying contrasting phenotypes: B7268 and NYH209. Whereas B7268 is highly resistant to metronidazole, highly adherent to host cells and very cytotoxic; NYH209 strain is sensible to metronidazole, poorly adherent and less cytotoxic to host cells [5]. Our transcriptome analysis identified a total of 25.105 and 24.956 genes expressed in B7268 and NYH209 strains, respectively. Principal component analysis (PCA) demonstrates that the transcriptome in replicates is more similar than the transcriptome between strains (PC1, 95% and PC2, 4% variance) (Fig. S1). While most of the genes were unchanged between strains, we identified 1.893 and 1.857 genes differentially expressed in B7268 and NYH209 strains, respectively (|log2-fold change| ≥ 1 and padj < 0.05) (Table 1S, Fig. 1A and 1B). Next, we investigated the enriched biological processes of differentially expressed genes (DEGs) in the B7268 and NYH209 strains through Gene Ontology (GO) analysis (Fig. 1C). Our results showed that genes upregulated in the highly adherent and resistant B7268 strain were associated to ribosome biogenesis, regulation of transcription, transmembrane transport, carbohydrate metabolic process, among others (Fig. 1C). Of particular interest, among the 25 genes most statistical significance and more expressed in B7268 cells several genes from protein families previously associated to parasite pathogenesis were identified; including 3 BspA family proteins (9.73, 6.64 and 6.63-fold), 2 glycoprotein 38 family (7.28 and 6.34-fold), a saposin B type domain (4.66-fold), 4 ribonuclease H protein family (8.28, 7.54, 5.40, and 5.01-fold), among others (Table 1). Additionally, genes previously associated with drug resistance [36] was also found among the 25 genes most differentially expressed in B7268, including 2 tryparedoxin peroxidase protein (5.21 and 4.46-fold), lactate dehydrogenase isozyme 2 (3.94-fold), intracellular auxin transport (4.96-fold) (Table 1). Collectively, these data identify a diverse set of upregulated genes that might be contributing, at least in part, to the observed effect in parasite drug resistance and/or enhanced adherence in B7268 strain. Alternatively, the differentially expressed genes identified in NYH209 were associated with transmembrane transporter activity, carbohydrate and amino acid metabolic process, DNA recombination, among others (Fig. 1C). The top 25 genes most differentially expressed in NYH209 cells include a transcription factor (5.46-fold), a ribosome biogenesis (7.10-fold), 2 cellular macromolecule catabolic process (9.40, and 7.15-fold) genes, a nitroreductase (10.11-fold), among others (Table 1).

### Chromatin accessibility in T. vaginalis strains

**Figure 2:**
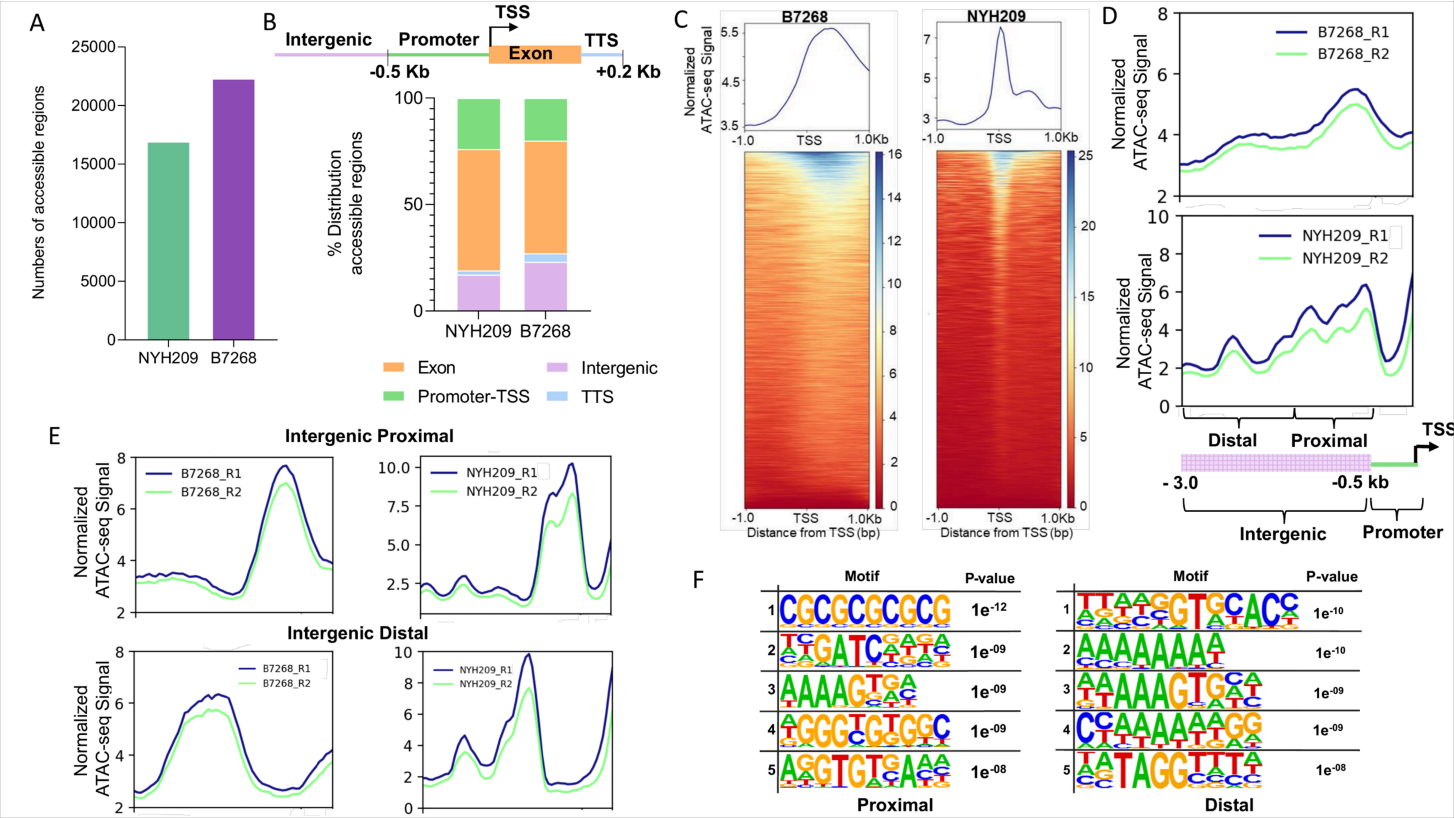
Overview of the ATAC-seq results. **(A)** Numbers of accessible regions detected in NYH209 (green) and B7268 strains (violet). **(B)** Genome distribution of accessible chromatin regions in *T. vaginalis* strains NYH209 and B7268. The stacked bar chart displays the percentage distribution of accessible regions across different genomic features: promoter-TSS (green), exons (orange), intergenic regions (pink), transcription termination sites (TTS, light blue). The majority of accessible regions are found in promoter-TSS and exon regions, with smaller contributions from intergenic regions and TTS. **(C)** Chromatin accessibility profiles of NYH209 and B7268 strains within a ± 1 kb from TSS. Heatmaps along with plot profiles of the averaged per-bin values across the scaled regions. **(D)** Chromatin accessibility profiles within a 3 kb window upstream of the TSS window and peak intensity at intergenic regions in NYH209 and B7268 strains. **(E)** Chromatin accessibility profiles regions within proximal (within 1.5 kb of the nearest gene, top) and distal (beyond 1.5 kb from the nearest gene, bottom) intergenic regions in B7268 and NYH209. The blue and green lines correspond to replicates 1 and 2, respectively. **(F)** Motif enrichment identified in proximal (left) and distal (right) intergenic regions conserved in NYH209 and B7268 strains.

To investigate the role of chromatin in gene expression regulation and assess differences in chromatin structure between strains, we conducted an assay for transposase-accessible chromatin (ATAC-seq) on the B7268 and NYH209 strains. The ATAC-seq data generated for each of these strains demonstrated that the similarity between replicates is high compared to the similarity between strains (Fig. S2). We identified 22.300 and 16.920 accessible regions in the B7268 and NYH209 strains, respectively (Fig. 2A, Table S2). We next examined the genomic distribution of open chromatin regions across genomic elements within each strain using the reference genome annotation (TrichDB-64_TvaginalisG32022) [25]. To this end, we defined four regions of interest: coding regions (named exon), intergenic regions, proximal promoters (defined as +/−500 bp from the TSS of each annotated gene) and TTS (which we defined as +/− 200 bp from the TTS of each annotated gene) (Fig. 2B). The distribution of ATAC-seq peaks across different genomic regions was generally consistent between both strains (Fig. 2B). In the B7268 strain, the analysis of genomic distribution revealed that 20% of the detected accessible chromatin regions were located in promoter regions, 53% were found within exons, while intergenic regions and transcription termination sites (TTS) accounted for 23% and 4%, respectively (Fig. 2B). In NYH209 strain, analysis of the genomic distribution showed that 24% of detected accessible chromatin regions covered promoters, 57% accessible chromatin were found in exons while intergenic regions and TTS account for 17% and 2%, respectively (Fig. 2B). When we examined the ATAC-seq signal upstream and downstream genes (± 1 kb), an enrichment of signal around Transcription Start Sites (TSS) was observed in both strains (Fig. 2C), corresponding to the expected location of an accessible promoter nucleosome depleted region. Interestingly, when examining the relationship between ATAC-seq peak distance and intensity at intergenic regions within a 3 kb window upstream of the TSS, we identified two regions of accessible chromatin in both analyzed strains: one located at 600 bp (proximal) and the other corresponding to 1200 bp from TSS (distal) in both analyzed strains (Fig. 2D and 2E) suggesting the presence of a regulatory sequence at those regions. Motif analysis of those distal and proximal chromatin accessible intergenic regions identified conserved motifs in both B7268 and NYH209 strains (Fig. 2F). The proximal motif (left panel) reveals a high level of conservation, particularly the CGGCGG sequence in the first motif, which may suggest a role in gene regulation near the transcription start site (TSS) (Fig. 2F). In contrast, the distal motifs (right panel) show enrichment for sequences such as GTGT and repeated A/T patterns, which are characteristic of regulatory elements potentially involved in long-range gene regulation (Fig. 2F). The presence of these conserved motifs in accessible intergenic regions across strains suggest potential functional relevance in modulating chromatin accessibility and transcriptional activity in *T. vaginalis*.

### Relationship between chromatin accessibility and the level of gene expression

**Figure 3:**
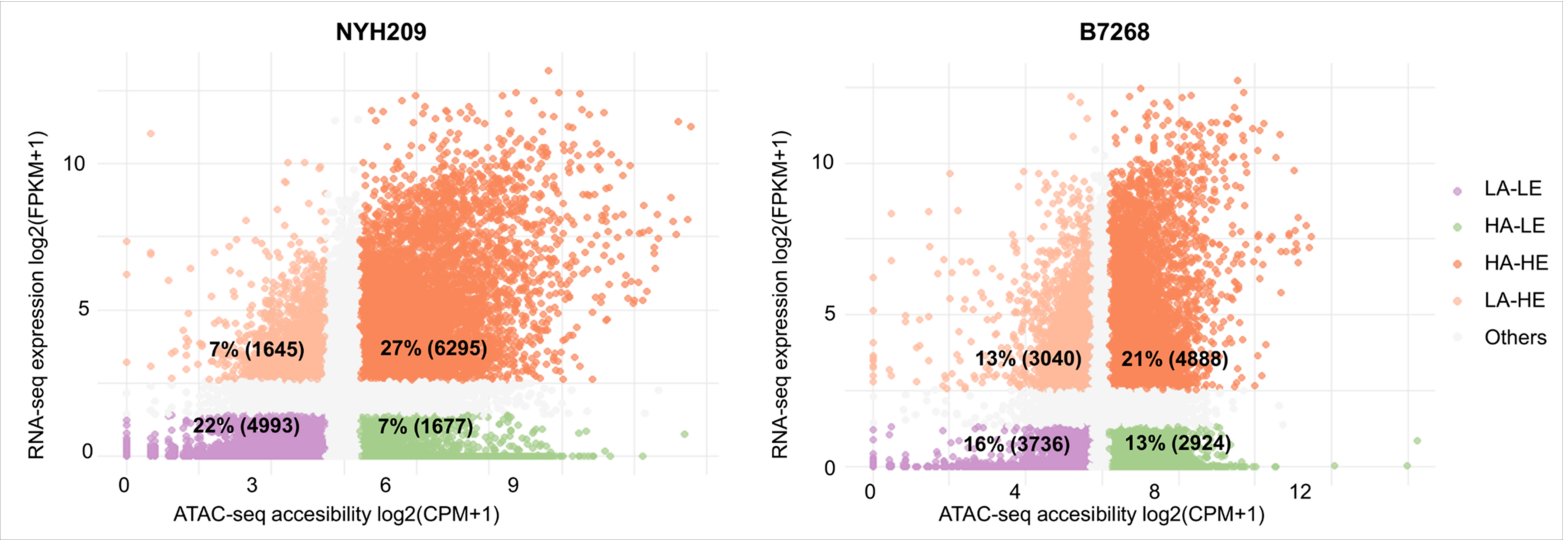
Integration ATAC-seq and RNA-seq show that promoter accessibility at the promoter and exon is strongly correlated with gene expression. Genes are grouped based on the level of promoter accessibility and gene expression. The number of genes in each group is shown in the parenthesis. LA: low accessibility, LE: low expression, HA: High accessibility, HE: High expression.

To evaluate the influence of chromatin accessibility in controlling the level of gene expression in *T. vaginalis*, we performed a co-analysis of RNA-seq and ATAC-seq data in both analyzed strains. To this end, we divided genes into four groups based on their level of expression and the level of promoter and exon accessibility. In the NYH209 strain, we observed a clear positive correlation between chromatin accessibility at promoter and exon regions and the transcript abundance of the corresponding genes (Fig. 3). Particularly, the majority of the analyzed genes exhibited both high accessibility and high expression (HA–HE: 6,295 genes), while the second largest group showed low accessibility and low expression (LA–LE: 4,993 genes) (Fig. 3). This correlation is also observed in the B7268 adherent strain where most of the genes presented high accessibility at the promoter and exon region and high expression (HA–HE: 4.888 genes), or low accessibility and low expression (LA–LE: 3.736 genes) (Fig. 3). These results clearly demonstrate that chromatin accessibility at the promoter/exon region is predictive of the gene expression level for a great number of genes in *T. vaginalis*. However, when we analyzed the intergenic regions and transcription termination sites (TTS), no correlation between accessibility and gene expression was detected on any of the analyzed strains (Fig. S3). It is important to mention, that we also observed group of genes displaying opposite patterns. One group of genes exhibiting high promoter/exon accessibility and low expression (HA-LE), and the other presenting low promoter/exon accessibility and high expression (LA-HE) (Fig. 3). Overall, these findings suggest that the relationship between promoter/exon accessibility and transcriptional activity appears to be strain-dependent. While a stronger correlation between accessibility and gene expression is observed in NYH209, another regulatory mechanisms seem to also play a role in B7268 strain.

### Chromatin accessibility and gene expression differences may explain strains phenotypes

**Figure 4:**
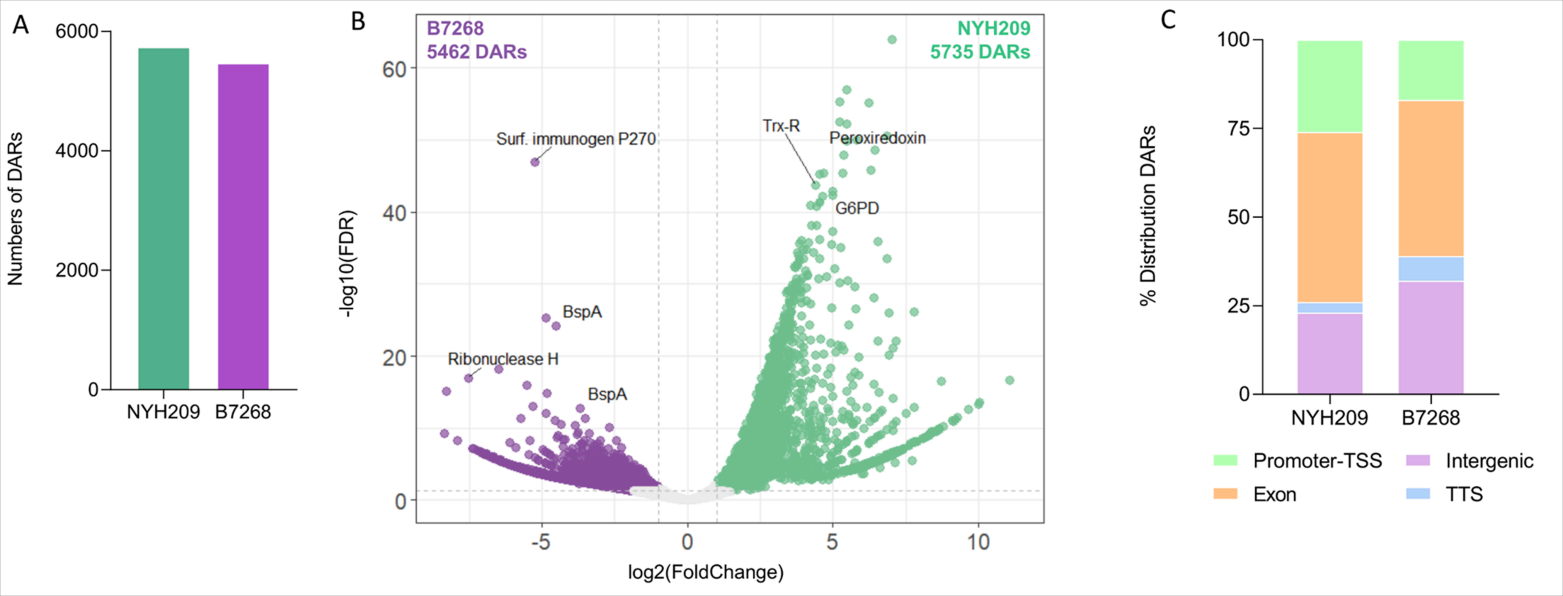
Analysis of differential accessible regions. **(A)** Numbers of differentially accessible regions (DARs) in NYH209 and B7268 strains identified by ATAC-seq. **(B)** Volcano plot showing DARs. The green dots represent DARs of NYH209 strain, the purple dots represent DARs of B7268 strain (|log2-fold change| ≥ 1 and FDR < 0.05), and the grey dots represent insignificant DARs identified from DiffBind-DESeq2. Genes highlighted in the plot include TVAGG3_0719780 (Ribonuclease H), TVAGG3_0339650 (Surf. Immunogen P270), TVAGG3_0794940 (BspA), TVAGG3_0766670 (BspA), TVAGG3_0344710 (Trx-R), TVAGG3_0380560 (G6PD), TVAGG3_0280800 (Peroxiredoxin). **(C)** Genomic distribution of DARs in NYH209 and B7268 strains. The stacked bar chart displays the percentage distribution of DARs across different genomic features: promoter-TSS (green), coding sequence/exons (orange), intergenic regions (pink), transcription termination sites (TTS, light blue).

Relatively little is known about specific mechanisms the parasite uses to regulate gene expression across different *T. vaginalis* strains. It is uncertain whether changes in chromatin organization can facilitate activation/repression of genes that could influence the phenotypic outcome of the strains. To assess whether chromatin accessibility influences the gene expression of adjacent genes, we first identified which specific genomic regions are differentially accessible between the B7268 and NYH209 strains. Our results indicate that chromatin accessibility is dynamic and varies between strains as 11.197 more accessible regions were identified between the B7268 and NYH209 strains (Table 3S, Fig. 4A and B). Specifically, 5.462 differentially accessible regions (hereafter referred to as DARs) were identified in B7268 and 5.735 DARs in NYH209 (Fig. 4B). The distribution of differential ATAC-seq peaks across different genomic regions was generally consistent between the two strains (Fig. 4C). In particular, B7268 showed that 17% of DARs are detected at promoters, 44% at exons, while intergenic regions and TTS accounted for 32% and 7% of DARs, respectively (Fig. 4C). In NYH209 strain, 23% of DARs covered promoters, 48% in exons, while intergenic regions and TTS account for 26% and 3% of DARs, respectively (Fig. 4C).

**Figure 5:**
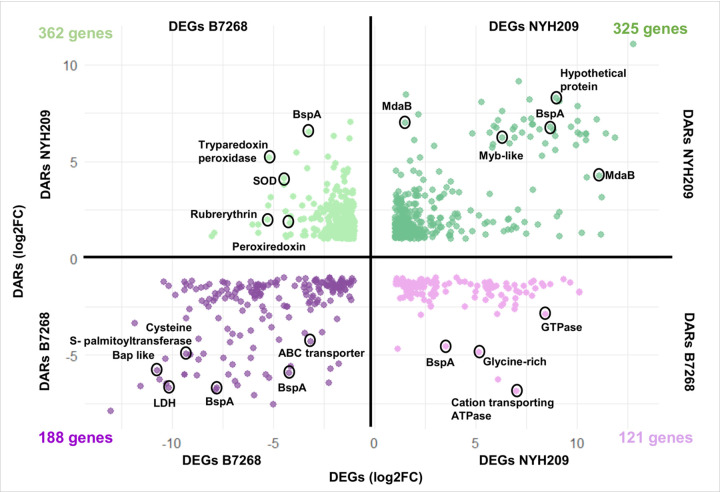
Integration DARs and DEGs. Comparison of Differentially Accessible Regions (DARs) and Differentially Expressed Genes (DEGs) in B7268 and NYH209 strains. Each point represents a gene, with the X-axes indicating changes in gene expression (log2FoldChange) and the Y-axes indicating changes in chromatin accessibility (log2FoldChange). A correlation is observed in both directions for the two strains, with a noticeable difference in the number of genes involved in each quadrant. Upper right quadrant (325 genes, green): genes DEGs with DARs in strain NYH209. Lower left quadrant (188 genes, purple): genes DEGs with DARs in B7268. Upper left quadrant (362 genes, light green): genes DEGs with less accessible chromatin in strain B7268. Lower right quadrant (121 genes, pink): genes DEGs with less accessible chromatin in strain NYH209.

**Figure 6:**
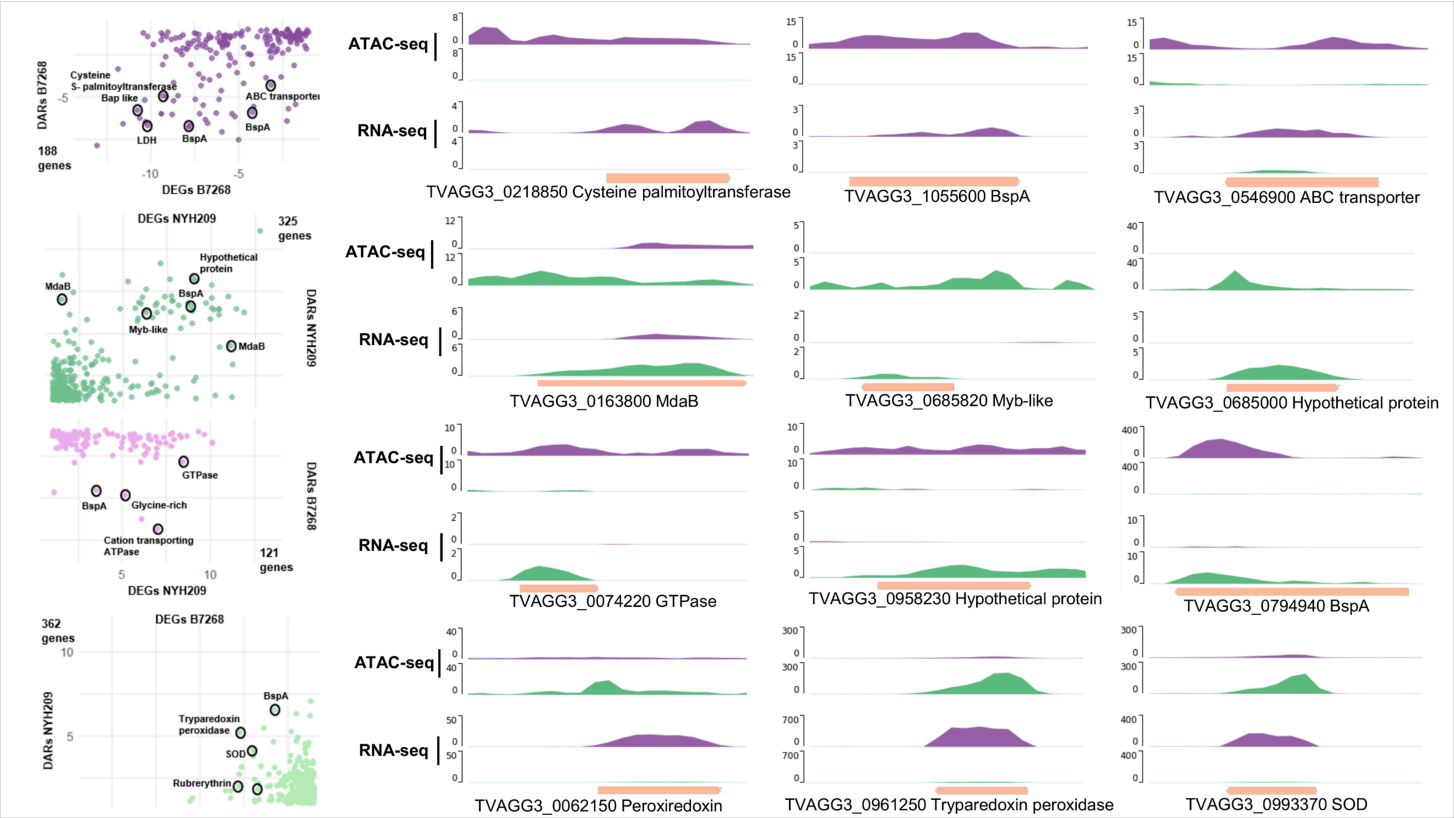
Integration DARs and DEGs. PyGenomeTracks screenshots of genomic regions (chromosomes: CM044617, CM044618, CM044619, CM044620, CM044621) displaying ATAC-Seq and RNA-Seq signals around 3 genes DEGs with DARs in B7268 (first row), genomic regions displaying 3 genes DEGs with DARs in NYH209 (second row),genomic regions displaying 3 genes DEGs in NYH209 with DARs in B7268 (third row), genomic regions displaying 3 genes DEGs in B7268 with DARs in NYH209 (bottom). Gene coding sequences are indicted as orange bars.

Then, we determined whether chromatin presenting DARs at promoters and/or exons might be modulating DEGs of their adjacent genes in each strain. In the B7268 strain, out of a total of 1893 differentially expressed genes (Fig. 1A), 188 were identified as having more accessible chromatin than NYH209 (Fig. 5). Similarly, of the 1,857 more expressed genes in the NYH209 strain (Fig. 1A), 325 were found to have more accessible chromatin than B7268 strain (Fig. 5). These results suggest that variations in chromatin accessibility may regulate the differential expression of specific genes observed on the parasite strains. Interestingly, our results showed that increased chromatin accessibility associated to high gene expression in B7268 strain was found in several genes that has been previously described to modulate parasite adherence [10, 13, 37, 38] (Fig. 5 and Fig. 6). Specifically, among the 188 DEGs having DARs in B7268, we identified 25 members of BspA family, 5 Bap-like genes, a cysteine S-palmitoyltransferase (TVAGG3_0218850), an Immuno-dominant variable surface antigen-like (TVAGG3_0661590), a Leishmanolysin-like metallopeptidase (TVAGG3_0411350), Ubiquitin hydrolase-like cysteine peptidase (TVAGG3_0290070) (Fig. 5 and Fig. 6). Additionally, we also identified genes related with metronidazole activation such as the modulator of drug activity b (NAD(P)H dehydrogenase (TVAGG3_0227060), a NADP-dependent oxidoreductase domain (TVAGG3_0679390), Thiol peroxidase (TVAGG3_0662620), Thioredoxin-like family (TVAGG3_0746620) and ABC transporter family protein (TVAGG3_0289470, TVAGG3_0546900 and TVAGG3_1031830) are more accessible and highly expressed in B7268 strain (Fig. 5). Additionally, among the 325 DEGs having DARs in NYH209, we also identified genes related to metronidazole pathway. Of particular interest, four MdaB genes including, NADPH-quinone reductase (TVAGG3_0325000), NADPH-dependent FMN reductase like (TVAGG3_0163800), NADP-dependent oxidoreductase domain (TVAGG3_0718940) and flavodoxin-like fold (TVAGG3_0585710) have been identified (Fig. 5 and Fig. 6). These findings suggest that genes associated with pathogenesis or the development of drug resistance in *T. vaginalis* appear to be regulated at the chromatin level.

Given that accessible chromatin might also negatively regulate gene expression, potentially by providing access to repressor complexes, we also analyzed the correlation between differentially accessible regions (DARs) and silenced genes in each strain (Fig. 6). Specifically, we identified 121 genes more expressed in NYH209 with less accessible chromatin compared to the B7268 strain. As example, genes such as cation-transporting E1-E2 ATPase (TVAGG3_0447130), structural constituent of cell wall (TVAGG3_0994440) and glycine-rich protein family (TVAGG3_0632810) demonstrated greater expression paired with lower chromatin accessibility in NYH209 (Fig. 6). This observation was particularly notable in the B7268 strain where we identified 362 differentially expressed genes with poorly accessible chromatin compared to the NYH209 strain (Fig. 6). Among them, we identified genes associated to metronidazole resistance such as tryparedoxin peroxidase (TVAGG3_0807190), peroxiredoxin protein (TVAGG3_0062150) or iron superoxide dismutase (TVAGG3_0993370) (Fig. 6). Together, these findings indicate that the accessibility of chromatin in the promoter region and/or gene body is not always associated with active transcriptional activity.

## Discussion

Early research on gene expression changes in *T. vaginalis* primarily relied on analysis of regulatory region located in gene promoters [11, 39, 40]. However, recent works highlighted the role of epigenetic mechanisms in regulating gene transcription in this parasite [12–15]. Considering this, we carried out an integrated analysis of genome-wide chromatin accessibility and transcriptome studies to characterize the role of chromatin accessibility controlling gene expression in two *T. vaginalis* strains: a highly adherent to host cells and metronidazole-resistant B7268 and a poorly adherent to host cells and metronidazole-sensitive strain NYH209. Previous results proposed that differences in gene expression likely contribute, al least partially, to the phenotypic variability observed between *T. vaginalis* strains [10]. In line with this, we identified a substantial number of differentially expressed genes between the B7268 and NYH209 strains, many of which have been previously linked to parasite pathogenesis. Notably, these include genes encoding BspA family proteins [37], Bap-like proteins [13, 41], and saposin-like proteins (SAPLIP) [42], among others. BspA protein family constitutes the largest gene family encoding potential extracellular proteins, which are likely to play a key role in modulating parasite-host cell adhesion [37, 38] and the differential expression of TvBspA genes under various growth conditions emphasize their functional diversity [37]. Similarly, members of the Bap-like protein family have also been linked to *T. vaginalis* pathogenesis as overexpression of TvBAP1 and TvBAP2 genes in a poorly adherent strain led to increased parasite attachment to human cervical epithelial cells [10]. We also identified the TvSaplip8 gene as differentially expressed in the adherent B7268 strain. SAPLIPs are recognized pore-proteins (PFPs) that play an essential to the pathogenicity of various protists, including *Entamoeba histolytica* and *Naegleria fowleri* [42]. These results are consistent with earlier research that identified TvSaplip12 as a cytopathogenic effector known for its hemolytic, cytotoxic, and bactericidal activities in *T. vaginalis* [42]. Based on the identification of genes previously associated to pathogenesis as highly expressed in the B7268 strain, we proposed that differential gene expression might contribute to parasite pathogenesis.

When evaluating the chromatin landscape, we noted that over half of accessible chromatin regions within the genome identified in both strains corresponded to promoter-proximal and coding regions, underscoring that a substantial portion of regulatory elements may occurs directly and/or very close to the associated genes. These findings align with the presence of core promoter elements, such as the DNA initiator (Inr), motifs 3 (M3) and 5 (M5), and the Myb binding site (MBS), located near the transcription start site of coding genes [39, 40, 43] as well as the previous definition of the minimal promoter necessary for efficient expression within the – 251 and – 51 relative to the transcription start site [44]. However, we have also identified open chromatin peaks residing at intergenic regions; suggesting the possibility that other distal regulatory elements may control gene expression. Specifically, we identified two chromatin accessible regions specifically located at –600 and – 1200 bp relative to the transcription start site. In concordance this, a previous report that analyzed the upstream regulatory sequences required for expression of the *T. vaginalis* α-succinyl CoA synthetase gene demonstrated that deletion of the 200 bp region from – 1051 to – 851 resulted in a drop-in the downstream gene activity compared to 53 ± 2% of the full-length construct, indicating the presence of positive regulatory elements in this region [44]. Additionally, the authors also found another region with positive regulatory elements located between – 651 and – 451, as deletion of these 200 bases results in a reduction of gene activity to 21 ± 8%. Presumably, these intergenic genomic regions harbor active cis-regulatory elements such as enhancers, insulators, or silencers. Further analysis is needed to demonstrate the role of these intergenic regions in modulation of genes expression in *T. vaginalis*.

Our results showed that there was a positive association between gene expression levels and chromatin accessibility at their promoter and/or gene coding regions in both analyzed strains. Importantly, we identified set of genes presenting increased expression and chromatin accessibly in the strain B7268 with a previously described function in adhesion to the host cell and/or metronidazole resistance [10, 13, 37, 38]. Of particular interest, we found that B7268 strain exhibits a distinct chromatin accessibility and higher expression in 25 genes with similarity to BspA [37, 38] and 5 Bap-like proteins [10, 13], known to mediate adherence to host cells. In agreement with this, our previous report demonstrated that the high expression of TvBAP1 and TVBAP2 is regulated by histone acetylation [13], which increase chromatin accessibility. In the current study, we also identified that the cysteine S-palmitoyltransferase gene exhibits high expression and chromatin accessibility in the adherent strain B7268. Supporting this notion of the requirement of protein palmitoylation in *T. vaginalis* pathogenesis, our earlier work showed that inhibiting palmitoylation with 2-bromopalmitate significantly reduces parasite aggregation and adhesion to host cells [45]. Finally, we detected a ubiquitin hydrolase-like cysteine peptidase (TVAGG3_0290070) and Leishmanolysin-like metallopeptidase (TVAGG3_0411350) as more expressed and chromatin accessible in B7268. Metallo and cysteine peptidases are responsible for more than half of proteolytic diversity in *T. vaginalis* [46]. Proteases from *T. vaginalis* have been shown to be significant modulators of pathogenesis, invasion, and survival [46]. The discovery of increased expression levels and enhanced accessibility of chromatin in genes associated with pathogenesis in the adherent strain B7268 underscores the crucial role of chromatin structure in modulating parasite pathogenicity. Our findings not only highlight the importance of chromatin accessibility in modulation of established pathways related to pathogenesis but also shed light on the significant role in driving the expression of key genes associated with metronidazole resistance in *T. vaginalis*. Drug resistance in eukaryotic microbes is a complex process and usually involves either drug activation/inactivation, or active drug efflux/reduced drug uptake [6, 47]. A central redox regulator with a pivotal role in the reduction of peroxiredoxin via thioredoxin are thioredoxin reductases (TrxR) [36]. In this context, our results showed that thioredoxin-like family protein (TVAGG3_0746620) exhibits high expression levels, accompanied by open chromatin, in the resistant strain B7268. Additionally, we have also identified additional genes potentially associated with drug resistance whose expression appears to be modulated at the chromatin level. These genes are highly expressed in the MTZ-resistant B7268 strain and may be subject to epigenetic regulation. Specifically, a Thiol peroxidase (Tpx, TVAGG3_0662620) and three ATP-binding cassette (ABC) transporter genes (TVAGG3_0289470, TVAGG3_0546900 and TVAGG3_1031830). Tpx is a peroxidase found in various eubacteria that exhibits H_2_O_2_-scavenging activity in the presence of thioredoxin and thioredoxin reductase [36, 48, 49]. ABC transporters are transmembrane proteins found in parasitic protozoans that play a key role in various cellular functions, including the mediation of drug transport away from their intended intracellular destinations [50]. In concordance with the results obtained here, a metal ABC transporter has been previously detected as overexpresses in a variety of MTZ-Resistant *T. vaginalis* strains [47]. Alternatively, four putative modulator of drug activity B (MdaB) genes are more expressed and accessible in the MTZ sensitive strain NYH209. Specifically, these MdaB genes are putative NADPH-dependent FMN reductase (TVAGG3_0163800), NAD(P)H-dependent oxidoreductase (TVAGG3_0718940), or NADPH-quinone reductase (TVAGG3_0325000) with flavodoxin domains and flavodoxin-like fold (TVAGG3_0585710). Our findings are in line with earlier research showing that MdaB genes are downregulated in strains of *T. vaginalis* that are drug resistant [47]. This suggests a potential regulatory mechanism involving chromatin accessibility influencing the expression of MdaB genes and implicates its role in mediating sensitivity to metronidazole treatment in *T. vaginalis*.

While open chromatin structure is associated with active gene transcription [51], DNA accessibility alone is not the primary determinant of gene regulation [52]. Even though many genes contributed to a positive correlation between chromatin accessibility and level of expression, an unexpected finding was the identification of genes with opposing patterns of accessibility and expression when combining the ATAC-seq and RNA-seq data. Specifically, we identified 121 and 362 genes higher expression in NYH209 and B7268 strain, respectively, that contain less accessible chromatin. This is consistent with recent studies challenging the traditional view that chromatin accessibility always correlates with active transcription. Others have shown that genes that are highly expressed might not be always easily accessible. Big regulatory complexes that bind to certain promoters may stop the transposase from gaining access to the DNA. In this sense, the EOR-1 transcription factor was discovered to occupy both accessible and inaccessible areas at its binding locations [53]. The low signal at the binding areas was thought to be caused by EOR-1 attaching close to nucleosomes or as a component of a larger complex that blocked the transposase ability to reach the DNA [53]. Along similar lines, it is also possible that large regulatory complexes formed by enhancer–promoter interactions would make the promoter less accessible to transposase cutting. Alternatively, inactive promoters can be accessible in some cells, suggesting transcription factors may bind without activating genes [52]. Repressed regions, like those indicated by H3K27me3, as well as active regions have an enrichment of ATAC-seq peaks [16, 17]. The discrepancy observed in some studies between chromatin accessibility and transcriptional activity in parasites may be attributed to the complexity of gene regulation in these organisms. While it has been suggested that accessible chromatin is associated with active transcription, experimental evidence shows that this relationship is not always straightforward. Factors such as genomic plasticity, complex interactions between regulatory proteins and chromatin elements, as well as variations in experimental conditions, can influence the interpretation of results.

## Conclusion

Our results indicate that the regulation of gene expression in *T. vaginalis* is a complex process, involving a nuanced interplay between chromatin accessibility and gene expression. Our study also provides insight into the comprehensive changes in chromatin accessibility that regulate the transcriptional outputs and signaling cascades, potentially influencing parasite pathogenesis or drug resistance. Fully understanding the mechanisms underlying gene expression regulation in *Trichomonas vaginalis* will require further investigation and integration of multidisciplinary data.

## Supplementary Material

Supplement 1

## Figures and Tables

**Table 1:** 25 genes most statistical significance and differentially expressed (DEGs) in B7268 and NYH209 identified by RNA-seq analysis.

GeneID	Product Description	log2FoldChange	padj
**25 more abundant genes in NYH209**			
TVAGG3_0783620	Ribosome biogenesis	7.10	1.09E-77
TVAGG3_0317350	Hypothetical protein	5.28	6.01E-64
TVAGG3_0341660	Malate dehydrogenase family	5.13	5.77E-52
TVAGG3_0002810	Amino acid transmembrane transporter protein	6.29	5.71E-48
TVAGG3_0430560	Glycoprotein 38 family	4.29	1.61E-45
TVAGG3_0839490	Cellular macromolecule catabolic process	7.15	5.74E-43
TVAGG3_0323520	Transcription factor	5.46	1.27E-40
TVAGG3_0306240	Winged helix DNA-binding domain family	5.70	3E-39
TVAGG3_0449180	LMO2823 protein family	3.96	1.43E-33
TVAGG3_0309560	Enolase	3.71	8.13E-33
TVAGG3_0959810	Nitroreductase family	10.11	6.15E-32
TVAGG3_0805200	Phosphoserine aminotransferase	3.95	2.91E-30
TVAGG3_0612090	Regulation of choline O-acetyltransferase protein	5.03	3.65E-30
TVAGG3_0319550	GTPase	3.84	7.76E-30
TVAGG3_0253000	Hypothetical protein	5.56	6.12E-29
TVAGG3_0300500	BspA family	4.84	3.56E-26
TVAGG3_0120480	Glucose import	3.12	6.51E-26
TVAGG3_0888510	Protein ubiquitination	4.92	1.19E-23
TVAGG3_0320000	Hypothetical protein	3.58	1.3E-23
TVAGG3_0308970	Cellular macromolecule catabolic process	9.40	1.8E-23
TVAGG3_0289530	L-fucose isomerase protein	3.87	1.21E-21
TVAGG3_0813330	Hypothetical protein	3.18	1.82E-21
TVAGG3_0031730	Alpha tubulin	2.92	2.55E-21
TVAGG3_0074000	Alpha tubulin	2.97	5.8E-21
TVAGG3_0529580	SEL-1-like family	3.81	1.4E-20
**25 more abundant genes in B7268**			
TVAGG3_0053460	Coronin family	−6.09	1.19E-89
TVAGG3_0664570	Ribonuclease H protein family	−8.28	6.21E-80
TVAGG3_0997820	Hypothetical protein	−5.67	1.72E-67
TVAGG3_0408780	Glycoprotein 38 family	−7.28	8.50E-53
TVAGG3_0266090	Glyceraldehyde-3-phosphate dehydrogenase	−6.05	5.61E-51
TVAGG3_0961250	Tryparedoxin peroxidase	−4.46	3.02E-50
TVAGG3_0387950	BspA family	−6.64	5.62E-50
TVAGG3_0387970	BspA family	−6.63	1.52E-49
TVAGG3_0349970	Rubrerythrin family	−5.28	1.71E-48
TVAGG3_0191610	BspA family	−9.73	1.54E-41
TVAGG3_0776680	Lactate dehydrogenase family protein	−3.94	5.44E-41
TVAGG3_0017540	Bifunctional inhibitor/lipid-transfer protein	−6.25	8.92E-40
TVAGG3_0858710	Ribonuclease H protein family	−5.40	2.34E-36
TVAGG3_0719780	Ribonuclease H-like family	−5.01	2.34E-36
TVAGG3_0385290	Ribonuclease H family	−7.54	5.17E-36
TVAGG3_0234670	SCP domain-containing protein	−4.85	1.77E-35
TVAGG3_0373890	Glycoprotein 38 family	−6.34	2.87E-35
TVAGG3_0061400	Saplip 8	−4.66	4.00E-35
TVAGG3_0471880	Hypothetical protein	−4.37	4.97E-35
TVAGG3_0183010	Barbed-end actin filament uncapping	−9.14	1.33E-34
TVAGG3_0974170	Intracellular auxin transport	−4.96	8.56E-32
TVAGG3_0807190	Tryparedoxin peroxidase	−5.21	1.63E-31
TVAGG3_0656230	Glucose-6-phosphate 1-dehydrogenase	−4.33	8.07E-31
TVAGG3_0278470	Hypothetical protein	−4.24	1.55E-30
TVAGG3_0410780	Hypothetical protein	−4.49	7.76E-30

## Data Availability

All data supporting the findings of this study are available within the paper and its Supplementary Information

## Abbreviations

ATAC-seq: Assay for Transposase-Accessible Chromatin using sequencing
DAR: Differentially Accessible Region
DEG: Differentially Expressed Gene
DNA: Deoxyribonucleic Acid
FPKM: Fragments Per Kilobase of exon per Million fragments mapped
HA: High Accessibility
HE: High Expression
HISAT2: Hierarchical Indexing for Spliced Alignment of Transcript 2
LA: Low Accessibility
LE: Low Expression
MDH: Malate Dehydrogenase
RNA-seq: RNA sequencing
TTS: Transcription Termination Site
TYM medium: Tryptase Yeast Extract Maltose medium
