# Supplementary material for: Chromatin accessibility and gene expression in the parasite Trichomonas vaginalis
Source: Res Sq. 2024 Dec 16:rs.3.rs-5455511. Preprint. [Version 1] doi: 10.21203/rs.3.rs-5455511/v1 (PMC11702783; doi:10.21203/rs.3.rs-5455511/v1)
Supplement: Supplement 1 [file NIHPPRS5455511v1-supplement-1.pdf]

Supplementary Files

This is a list of supplementary files associated with this preprint. Click to download.

- supplementaryFiguresandTableslegends.docx
- FigureS1.tiff
- FigureS2.tiff
- FigureS3.tiff
- Supplementarytable1.xlsx
- Supplementarytable2.xlsx
- Supplementarytable3.xlsx
- Supplementarytable4.xlsx
